# Functional Analysis of Retinal Flecks in Stargardt Disease

**DOI:** 10.4172/2155-9570.1000233

**Published:** 2012-07-30

**Authors:** Tommaso Verdina, Stephen H. Tsang, Vivienne C. Greenstein, Jana Zernant, Andrea Sodi, Luiz H. Lima, Stanley Chang, Rando Allikmets, Ugo Menchini

**Affiliations:** 1 Department of Specialized Surgical Sciences, Eye Clinic, University of Florence, Florence, Italy; 2 Department of Ophthalmology, Columbia University, New York, NY, USA; 3 Department of Pathology and Cell Biology, Columbia University, New York, NY, USA; 4 Department of Ophthalmology, Federal University of Sao Paulo, Sao Paulo, Brazil

**Keywords:** Stargardt disease, Fundus flavimaculatus, Flecks, Fundus autofluorescence, Microperimetry, Spectral domain optical coherence tomography

## Abstract

**Purpose:**

To evaluate visual function of flecked areas in a series of patients with Stargardt disease (STGD) and compare them with adjacent non flecked areas.

**Methods:**

Twenty–seven patients with STGD, ABCA4 mutations and yellowish retinal flecks at fundus examination were recruited. Microperimetry with the Nidek MP-1 and fundus autofluorescence imaging (FAF) were performed in all the patients (27 eyes) while spectral-domain optical coherence tomography (SD-OCT) was performed in a subgroup of patients (20 eyes). Visual sensitivity (in dB) for each hyperfluorescent flecked area on FAF was compared with the value of the nearest adjacent non-flecked area in the MP-1 grid and at approximately the same distance from the fovea. Retinal structure in some of the flecked areas tested by microperimetry was analysed with SD-OCT. All patients were screened for mutations in the ABCA4 gene by APEX array and direct sequencing.

**Results:**

A total of 1836 locations (68 locations for each eye with the 10-2 program) were tested with the MP-1 and 97 corresponded to hyperautofluorescent flecks. A repeated measure, linear regression analysis was used to evaluate differences between visual sensitivity associated with the 97 flecked areas with those in the 97 neighbouring non-flecked areas. The difference was statistically significant (p<0.001) (flecked areas 12.89 +/− 3.86 dB vs. non-flecked areas 14.40 +/− 3.53 dB, respectively). SD-OCT in the flecked areas revealed the presence of hyperreflective dome-shaped lesions in the outer retina located at the level of the retinal pigment epithelium (RPE), with dislocation or disruption of the photoreceptor layer.

**Conclusions:**

In STGD hyperfluorescent flecks on FAF are associated with decreased visual sensitivity compared to adjacent non-flecked areas and with an alteration of the photoreceptor layer on OCT. Flecks do not represent only a typical ophthalmoscopic feature but correspond, in some cases, to retinal damage that contributes to patients’ visual loss.

## Introduction

Stargardt disease (STGD) is an inherited macular dystrophy characterized by infantile onset and progressive loss of central visual function [1,2] and is usually inherited as an autosomal recessive trait. STGD is caused by mutations in the ABCA4 gene coding for a transport protein that is involved in the visual cycle and located in the photoreceptor outer segments [3–6]. The exact sequence of the disease process for STGD is not completely understood, however the generally accepted hypothesis suggests that defective transport of vitamin A derivatives due to mutant ABCA4 protein results in abnormal accumulation of visual cycle by-products (bis-retinoids, lipofuscin) in the retinal pigment epithelium (RPE) [7] with consequent RPE degeneration and photoreceptor disruption [8–11].

On fundus examination, macular atrophy is often associated with typical fish-tail white-yellowish spots (flecks) at the posterior pole and sometimes at the retinal midperiphery. These flecks vary in size and shape, for example they can be small or large, appear round, fusiform, pisciform, or X shaped. They have a yellow-whitish colour and are well defined at the early stages of the disease process. They often become hazy, grey, ill defined and barely detectable on fundus examination. However the flecks are clearly evident on fundus autofluorescence (FAF) as hyperfluorescent or sometimes hypofluorescent areas in the later stages of the disease. In the past the presence of flecks was considered a distinguishing characteristic of the disease “fundus flavimaculatus” which could be associated with atrophic maculopathy. Currently macular atrophy and fundus flavimaculatus (and their possible association) are considered variants of the same disease.

It has been suggested that flecks’ hyperautofluorescence may represent a precursor of photoreceptor death and RPE atrophy [12–15]. In a previous study [16] of seven STGD patients it was found that hyperfluorescent flecks on FAF were not associated with decreased visual sensitivity. The aim of our study was to compare the functional features of flecked and adjacent non-flecked areas using microperimetry (MP-1) and fundus autofluorescence (FAF). Spectral-domain optical coherence tomography (SD-OCT Cirrus or 3D-Topcon 1000) was performed in the flecked areas in a subgroup of patients to investigate the structural characteristics.

## Methods

Patients diagnosed with STGD and with flecks at the posterior pole were identified retrospectively at the Eye Clinic, University of Florence, Italy and at the Edward S. Harkness Eye Institute of Columbia Medical Center in New York City, NY. The medical records and imaging studies of 27 consecutive patients were retrospectively reviewed according to the guidelines of the local Ethical Committees at the University of Florence and at Columbia University.

The criteria for STGD phenotype included the following: appearance in the first or second decade of life; bilateral progressive central vision loss; macular atrophy/dystrophy; normal caliber of retinal vessels; absence of pigmented bone spicules; and normal or mildly abnormal full-field electroretinogram results.

Patients with refractive errors (>+/− 5D), significant cataract, other ocular diseases, pupil diameter after dilation <4 mm, age >70 years, and previous photorefractive treatment were excluded from the study. Moreover none of the study patients had a family history of other inherited retinal or systemic disorders. All patients included in the study had mutations in the ABCA4 gene. The 27 patients underwent a complete ophthalmic examination, color fundus photography, microperimetry and FAF. Twenty patients were examined with SD-OCT.

Microperimetry was performed with the MP-1 (Nidek Technologies, Padova, Italy) following pupil dilation with 0.5% tropicamide and 2.5% phenylephrine and a period of adaptation of 30 minutes to dim room illumination. A 10-2 pattern with 68 locations was used to assess sensitivity in the macular area. “White” test lights (stimulus size Goldmann III, 200 ms in duration) were presented on a dim “white” background (1.27 cd/m2) using a 4-2 procedure. The patient was asked to maintain fixation on a 2° red cross during the test and the non-tested eye was occluded. In addition to recording the sensitivity at each test location the stability and location of fixation during testing were also recorded. Stability was defined both in terms of the percentage of fixation points that fell within a 2° and 4° diameter circle during the visual field test and in terms of the bivariate contour ellipse area (BCEA) as previously described [11,17]. All patients had prior experience of MP-1 tests of visual function.

Fundus autofluorescence (FAF) imaging was performed with a confocal scanning laser ophthalmoscope (Heidelberg Retina Angiograph 2; Heidelberg Engineering, Dossenheim, Germany) using a 30° field of view at a resolution of 1536 × 1536 pixels. An optically pumped solid-state laser (488 nm) was used for excitation and a 495 nm barrier filter was used to modulate the blue argon excitation light. Standard procedure was followed for the acquisition of FAF images, including focus of the retinal image in the infrared reflection mode at 820 nm, sensitivity adjustment at 488 nm, and acquisition of 9 single 30° × 30° FAF images encompassing the entire macular area with at least a portion of the optic disc. The 9 single images were computationally averaged to produce a single frame with improved signal-to-noise ratio.

FAF imaging was always performed after MP-1 examination to avoid the possible effect of light adaptation on measures of visual sensitivity.

**Figure 1:**
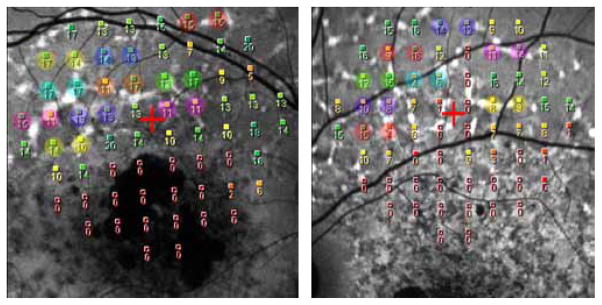
Figure 1a–1b: MP-1 sensitivity grid with superimposed FAF image in the NAVIS software in patients ID 17 and 18. Comparison of visual sensitivity (in dB) of flecked areas with the nearest non-flecked areas. Visual sensitivity of the locations encircled by the same colour has been compared to each other.

The 30° FAF images were imported into the NAVIS software in the Nidek MP-1 system and overlaid on the MP-1 results using retinal vessel bifurcation as registration landmarks. Hyperfluorescent flecks were carefully identified and for each patient the eye that had the most flecks associated with MP-1 test locations was selected for analysis. Flecked areas were included in the study either if the MP-1 test location was exactly over the fleck or at the margin of it. Visual sensitivity (in dB) for each flecked area was compared with that of an adjacent non flecked area in the MP-1 grid at a distance of 2°. The areas were outside the macular atrophic area outlined by FAF imaging (Figure 1). With this procedure visual sensitivities in areas with or without flecks at approximately the same distance from the fovea were evaluated. The location of the foveal center was determined by using visible landmarks such as perifoveal capillaries or sometimes the presence of xanthophyl.

Statistical analysis was performed using SPSS (Statistical Package for Social Sciences Inc., Chicago, IL, USA) software for Windows (Version 18.0). In order to compare the sensitivity values for flecked and non-flecked areas a repeated measures linear regression analysis was used. A p-value <0.05 was considered to indicate statistical significance.

SD-OCT was performed in a sub-group of 20 patients on the flecked areas that were evaluated with the MP-1. Cirrus Spectral Domain OCT (Carl Zeiss Meditec Inc., Dublin, CA, USA) was used for 12 patients and Topcon 3D-OCT 1000 (Topcon Inc., Paramus, New Jersey, USA) for 8 patients. Both instruments allow for simultaneous OCT scans and fundus photograph and subsequent image superimposition. The acquisition protocol consisted of a macular cube 512 × 128 scan pattern in which a 6.0 × 6.0 mm region of the retina was scanned (a total of 65,536 sampled points) within a scan time of 2.4 seconds. After image acquisition, those with a signal strength ≤ 8 were excluded. The precise location and orientation of each scan were determined using the simultaneous OCT grey-scale imaging. The morphology of the flecks and their location within the retina were analysed; particular attention being paid to the presence of abnormalities in the inner segment ellipsoid (ISe) band [18] located above the lesion. Each scan was independently evaluated by three different observers ( TV, AS and UM ) who classified the identified flecks into two groups of structural abnormalities: i.e. for some flecks the ISe was still present but it was dislocated upward, while for other flecks the ISe was more severely disrupted. In cases of disagreement between the observers the opinion of the senior observer (UM) was accepted. The non-parametric Mann-Whitney test for unpaired data was used for comparisons between MP-1 sensitivity of flecks associated either with ISe dislocation or with ISe disruption and the nearest non-flecked area at the same distance from the fovea. A p-value <0.05 was considered to indicate statistical significance.

Genetic analysis was performed on all patients by a combination of ABCR500 microarray [19] and direct sequencing, including the Next Generation Sequencing (NGS) approach [20].

## Results

Twenty–seven eyes from 27 patients (9 males and 18 females, 15 from the Eye Clinic in Florence and 12 from Columbia University in New York) with STGD and ABCA4 mutations were included in the study. The average age was 40.74 +/− 14.86 (range 22–68 yrs.). Best corrected visual acuities (BCVA) ranged from 20/40 to 20/320.

The results of the MP-1 showed that the preferred retinal location (PRL) was foveal in 4 eyes (14.8%) and extrafoveal in 23 (85.2%). The PRL was superior in 20 eyes, inferior in 1, temporal in 1 and nasal in 1. Fixation was classified as stable in 4 eyes, relatively unstable in 16, and unstable in 7. In terms of BCEA the mean 68.2% fixation value was 8.40 +/− 6.92°^2^ while the mean 99% value was 35.07 +/− 35.17°^2^.

The eccentric PRLs for the 23 eyes were located on average 5.87° from the fovea (ranging from 1.9° to 12.8°) and in 16 of the 23 eyes (69.6 %) the PRL was located at a significant distance from the edge of the macular atrophy.

A total of 1836 locations (68 locations for each eye with the 10-2 program) were tested with the MP-1. The location of the test light was associated with 97 hyperfluorescent flecked areas: the mean retinal sensitivity was 12.89 +/− 3.86 dB. In the 97 neighbouring non-flecked areas that we considered for the comparison, the mean sensitivity was 14.40 +/− 3.53 dB. To determine whether the difference in sensitivity in dB between flecked and non-flecked areas was significant a linear regression model for repeated measurements was conducted. The mean difference between flecked and non-flecked areas [geometric mean 1.15 dB (95% CI −1.21; 1.08)] was statistically significant (p< 0.001).

**Figure 2:**
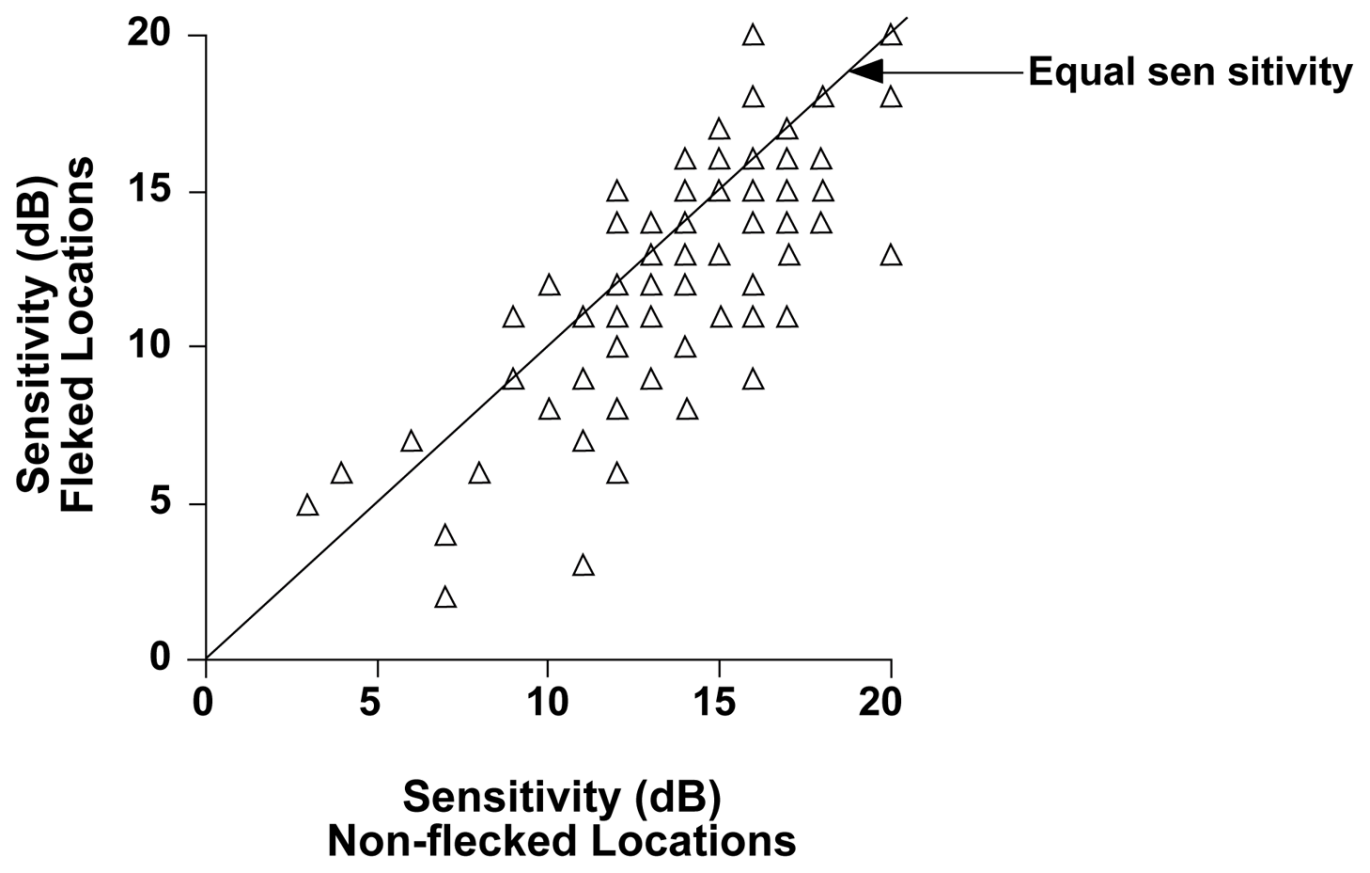
Sensitivity of flecked areas to the adjacent non-flecked areas. The points that fall below the 45° line represent flecked areas that have lower sensitivity than the corresponding non-flecked area.

Figure 1 and Figure 2 show the comparison of the sensitivity of each flecked area to the nearest non-flecked area at the same distance from the fovea; 52 flecked areas were associated with a decrease in sensitivity compared to adjacent non flecked areas, 34 had equal sensitivity (by equal sensitivity we mean equal dB values or a difference of +/− 1 dB) and only 11 flecked locations were associated with a increase in sensitivity.

**Figure 3:**
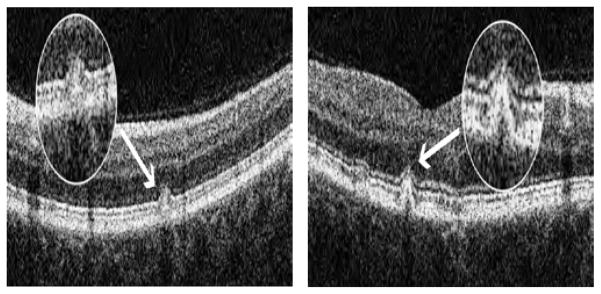
Figure 3a–3b: Dislocation of the ISe band on SD-OCT associated with smaller sized flecks and with no significant decrease in visual sensitivity (patients ID 24 and 20).

**Figure 4:**
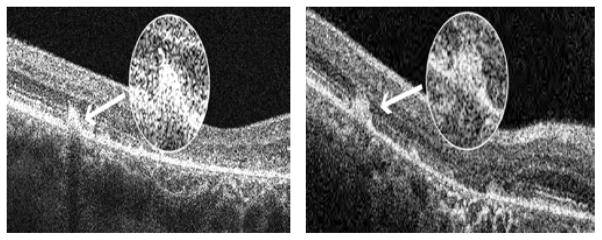
Figure 4a–4b: Disruption of the hyper reflective line corresponding to the ISe band on SD-OCT associated with a bigger accumulation of lipofuscin and with significant decreased visual sensitivity (patients ID 4 and 25).

The flecked areas of 20 of the patients evaluated by MP-1 were examined with SD-OCT. The SD-OCT scans revealed the presence of hyperreflective dome-shaped lesions in the outer retina located at the level of the RPE. Some flecks appeared to lift up or elevate the hyperreflective line corresponding to the ISe band (Figure 3a–3b) while other flecks were associated with a disruption of the ISe band (Figure 4a–4b)

**Figure 5:**
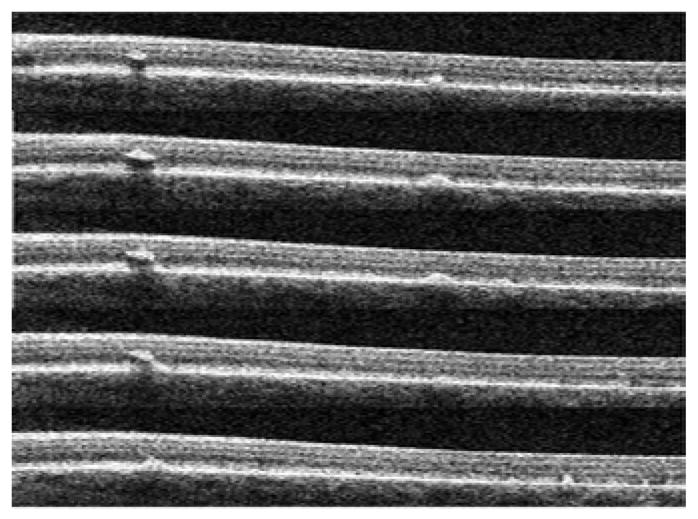
Consecutive SD-OCT line scans in macular cube mode demonstrating a connection between an apparent isolated fleck located in the ONL and the RPE (patient ID 4).

In some cases these hyperreflective lesions seen on SD-OCT were located in the outer nuclear layer (ONL) and seemed to be separated from the RPE. An analysis of consecutive line scans performed by means of the SD-OCT with macular cube mode demonstrated that these hyperreflective lesions were in a continuum with the inner part of the RPE layer in the form of a bridge of hyperreflective material connecting these apparently isolated alterations with the RPE (Figure 5).

Among the 67 flecked areas analysed by SD-OCT, 49 were associated with a dislocated but intact ISe band, while in 18 flecked areas the ISe band was disrupted.

On average the visual sensitivity of flecked areas associated with disruption of the ISe band was significantly decreased (p=0.01) in comparison with that of the nearest non-flecked area at the same distance from the fovea (flecked areas with disrupted or absent ISe band 8.33 +/− 2.59 dB vs. adjacent non-flecked areas 11.11 +/− 3.36 dB, respectively), while in the flecked areas where the ISe band was dislocated upward but intact, the difference was not statistically significant (flecked areas with intact ISe band 14.53 +/− 2.51 dB vs. adjacent non-flecked areas 15.10 +/− 2.79 dB, respectively with p=0.1).

## Discussion

In this retrospective study of STGD patients designed to investigate functional features associated with hyperfluorescent flecks seen on FAF, we found that visual sensitivity was significantly decreased in flecked areas compared to neighbouring non-flecked areas.

On SD-OCT the flecked areas corresponded to hyperreflective lesions at the level of the RPE associated with a dislocation or disruption of the ISe band [21,22].

The RPE and photoreceptor abnormalities shown by our morphological investigations with SD-OCT are consistent with previous histopathologic reports of lipofuscin accumulation within the RPE and photoreceptor loss in STGD [9–11].

Recently Voigt et al. [23] proposed a classification for retinal flecks in fundus flavimaculatus based on SD-OCT findings: the classifications comprise 5 distinct types of lesion in relation to their localization in the outer retinal layers. In our series we never observed isolated small hyperreflective deposits located at the level of the outer nuclear layer (ONL) clearly separated from the RPE. The macular cube scan performed with our SD-OCT allowed us to carefully check the morphology of flecks lesions. For the few cases where we found a lipofuscin accumulation within the ONL, we always observed an anatomical connection of these apparently isolated flecked lesions with the underlying RPE (Figure 5). This suggests that the alteration and the accumulation of lipofuscin initially starts in the RPE and then expands to inner retinal layers. We can speculate that the different flecks types described by Voigt et al. simply represent different appearance and stages of the same kind of lesions, as suggested by the authors themselves. Of note, we did not perform a systematic SD-OCT analysis of all the flecks that were identified, but considered only the SD-OCT scans representing flecks whose sensitivities were assessed with the MP-1.

Regarding the appearance of the ISe band above the fleck lesion, we observed that this varied. In some cases the band was simply displaced upwards (Figure 3a–3b) while in other cases, it was disrupted or completely absent (Figure 4a–4b); this latter was associated with flecks with significantly decreased sensitivity in comparison to adjacent nonflecked areas.

Our results suggest that visual function in flecked areas may be related to the severity of the underlying structural abnormalities: i.e. a disruption of the ISe band is associated with significant anatomical and functional damage while a displacement appears to result in less severe functional deficits.

We are aware that there are some limitations in our study. MP-1 values can be variable in patients with unstable fixation even with the eye-tracking system. In addition the overlapping of FAF and MP-1 sensitivity maps may not be perfect and the comparison of two adjacent retinal locations may not be exactly at equal distances from the fovea.

In conclusion, we found that hyperautofluorescent retinal flecks are associated with a statistically significant reduction of visual sensitivity on the MP-1 and with an alteration of the photoreceptor layer as seen on SD-OCT. In the flecked areas where the ISe band is disrupted or completely absent the functional alterations are probably irreversible. Hence, flecks do not represent only a typical ophthalmoscopic feature useful for diagnosing and monitoring the disease but, in some instances, correspond to retinal damage that contributes to patients’ visual loss in STGD.

## Summary

Fundus autofluorescence (FAF), microperimetric (MP-1) and spectral-domain optical coherence tomography (SD-OCT) findings in the flecked areas of the retina of a series of Stargardt disease (STGD) patients are described. These imaging modalities are useful non-invasive techniques that can aid in the diagnosis of STGD and contribute to our understanding of its pathophysiology.

## Figures and Tables

**Table 1:** Clinical and Genetic characteristics.

ID	Initials	Age	Sex	Eye	BCVA	Genetics
1	AA	33	F	OS	0.4	c.5882G>A (p.Gly1961Glu); c.2382+1G>A
2	AN	39	F	OD	0.5	c.6320G>A (p.Arg2107His); c.3523−1G>A
3	ERA	26	F	OD	0.3	c.4139C>T (p.Pro1380Leu); c.4139C>T (p.Pro1380Leu)
4	FP	44	M	OS	1	NS - not screened
5	GS	59	M	OD	0.7	c.4139C>T (p.Pro1380Leu); c.5087G>A (p.Ser1696Asn)
6	HR	48	M	OS	0.3	c.318T>G (p.Y106*); c.2588G>C (p.Gly863Ala)
7	MC	24	F	OD	1	c.1622T>C (p.Leu541Pro)/c.3113C>T (p.Ala1038Val)
8	MCH	65	F	OD	1.3	c.4594G>A (p.Asp1532Asn); c.5882G>A (p.Gly1961Glu)
9	PS	28	F	OD	1.3	c.5882G>A (p.Gly1961Glu); IVS43+1G>T
10	SK	68	F	OD	0.6	c.5882G>A (p.Gly1961Glu); c.5882G>A (p.Gly1961Glu)
11	RE	47	M	OS	0.8	c.1622T>C (p.Leu541Pro)/c.3113C>T (p.Ala1038Val)
12	WR	34	F	OD	0.7	c.5882G>A (p.Gly1961Glu)
13	BJ	23	F	OS	1	c.4667+1G>A; c.5512C>A (p.His1838Asn)
14	BC	42	F	OD	0.7	c.2461T>A (p.Trp821Arg); c.5714+5G>A
15	CS	38	F	OS	1.1	c.634C>T (p.Arg212Cys); c.3056C>T (p.Thr1019Met)
16	DG	63	F	OS	0.1	c.1714C>T (p.Arg572*); c.4417C>A (p.Leu1473Met)
17	DC	28	F	OS	0.7	c.5882G>A (p.Gly1961Glu); c.3233G>A (p.Gly1078Glu)
18	FA	39	M	OD	1	c.2345G>A (p.Trp782*); c.6320G>A (p.Arg2107His)
19	GD	68	M	OD	0.9	c.4297G>A (p.Val1433Ile); c.4297G>A (p.Val1433Ile)
20	GPA	43	M	OD	0.1	c.3610G>A (p.Asp1204Asn); c.5527C>T (p.Arg1843Trp); c.6079C>T (p.Leu2027Phe)
21	MS	28	F	OD	0.7	c.3323G>A (p.Arg1108His); c.4297G>A (p.Val1433Ile)
22	NF	39	F	OD	0.7	c.634C>T (p.Arg212Cys); c.5087G>A (p.Ser1696Asn)
23	OA	29	F	OS	0.7	c.768G>T (p.Val256Val); c.5714+5G>A
24	PE	22	M	OD	1	c.4383G>A (p.Trp1461*); c.5929G>A (p.Gly1977Ser)
25	SG	64	F	OD	1.1	c.5882G>A (p.Gly1961Glu); c.571−2A>T
26	TM	33	M	OS	0.6	c.2461T>A (p.Trp821Arg); c.2461T>A (p.Trp821Arg)
27	TS	26	F	OS	1	c.6537del (p.Pro2180Leu fs*3)
